# Electrospun Single Crystalline Fork-Like K_2_V_8_O_21_ as High-Performance Cathode Materials for Lithium-Ion Batteries

**DOI:** 10.3389/fchem.2018.00195

**Published:** 2018-06-01

**Authors:** Pengfei Hao, Ting Zhu, Qiong Su, Jiande Lin, Rong Cui, Xinxin Cao, Yaping Wang, Anqiang Pan

**Affiliations:** Department of Materials Physics, School of Materials Science and Engineering, Central South University, Changsha, China

**Keywords:** potassium vanadate, electrospinning, fork-like nanostructure, cathode materials, lithium-ion batteries

## Abstract

Single crystalline fork-like potassium vanadate (K_2_V_8_O_21_) has been successfully prepared by electrospinning method with a subsequent annealing process. The as-obtained K_2_V_8_O_21_ forks show a unique layer-by-layer stacked structure. When used as cathode materials for lithium-ion batteries, the as-prepared fork-like materials exhibit high specific discharge capacity and excellent cyclic stability. High specific discharge capacities of 200.2 and 131.5 mA h g^−1^ can be delivered at the current densities of 50 and 500 mA g^−1^, respectively. Furthermore, the K_2_V_8_O_21_ electrode exhibits excellent long-term cycling stability which maintains a capacity of 108.3 mA h g^−1^ after 300 cycles at 500 mA g^−1^ with a fading rate of only 0.043% per cycle. The results demonstrate their potential applications in next-generation high-performance lithium-ion batteries.

## Introduction

Rechargeable lithium-ion batteries (LIBs) are one of the most important energy storage devices in microchips, cell phones, electric vehicles (EVs), and hybrid electric vehicles (HEVs) (Whittingham, 2004; Armand and Tarascon, 2008; Zheng et al., 2015; Ou et al., 2017; Pan et al., 2017a,b; Yang et al., 2017). Layer-structured LiCoO_2_ has been extensively studied and largely used as commercial cathode materials due to their high working potential, high energy density and good cycling performance. However, it delivers a relatively low capacity of ~130 m Ah g^−1^, which is only half of its theoretical capacity, thus restricting its further expansion in LIBs (Goodenough and Kim, 2010; Liang et al., 2014). In addition, the scarcity of Co resources has further pushed up the cost of LIBs manufacturing. Therefore, it would be interesting to find alternative electrode materials with lower cost, larger specific capacity and better safety.

As important cathode candidates in LIBs, vanadium oxides and vanadate have attracted extensive interests owing to their abundant reservation, high specific capacity, and high Li^+^ diffusion efficiency (Maingot et al., 1993; Torardi and Miao, 2002; Ng et al., 2007; Liu et al., 2009; Mai et al., 2010a,b; Wee et al., 2010; Liu and Yang, 2011; Pan et al., 2011a; Rui et al., 2011; Varadaraajan et al., 2011; Wang et al., 2011; Liang et al., 2013b; Jian et al., 2014; Zhou et al., 2014; Meng et al., 2016; An et al., 2017). For example, vanadium pentoxide (V_2_O_5_) has a high theoretical capacity of 442 mA h g^−1^, but its commercialization is still limited by poor cyclic stability (Owens et al., 1999; Cao et al., 2005; Wang et al., 2006). Many studies have demonstrated that the incorporation of metal cations (such as Li^+^, Na^+^, K^+^, Ag^+^, Zn^2+^, Cu^2+^, etc.) (Ma et al., 2008; Liu and Tang, 2009; Cheng and Chen, 2011; Liang et al., 2013a, 2014; Bach et al., 2014; Yang et al., 2016) into V_2_O_5_ interlayers is favorable to improve the electronic conductivity (Zhou et al., 2014), simultaneously creating more Li^+^ intercalation channels (Khoo et al., 2010) and improve the structural stability (Pan et al., 2011b). It was reported that introduction of K^+^ into vanadium oxygen layers is effective to deliver high specific capacity and excellent reversibility by the pillar effect and larger interlamellar space (Baddour-Hadjean et al., 2011, 2014; Xu et al., 2012, 2015; Fang et al., 2015, 2016; Meng et al., 2016). In addition, K_2_V_8_O_21_ is a more stable crystal phase compared to V_2_O_5_ or KVO_3_ and it has a higher theoretical capacity of 261 mA h g^−1^. Manev et al. reported the usage of K_2_V_8_O_21_ as a cathode material for the first time, which deliver an initial discharge capacity of about 190 mA h g^−1^, but with a poor capacity retention (Manev et al., 1993). The electrochemical performance of K_2_V_8_O_21_ can be improved by incorporation water into the layered structure by hydrothermal treatment (Aleksandrova et al., 2006, 2009). By doping inactive metal ions such as Nb^5+^ and Ti^4+^ into the crystal structure by mechanical ball milling, the capacity of K_2_V_8_O_21_ (Ni et al., 2015) can also be enhanced. However, the cycling performance of K_2_V_8_O_21_ is still unsatisfactory, in particular for the long-term cycling stability.

One-dimensional (1D) nanostructures such as nanowires (Mai et al., 2010b), nanorods (Gu et al., 2015), nanofibers (Wee et al., 2010) could offer shorter Li-ion diffusion pathways, higher specific surface area and faster electron transfer along longitudinal direction (Li et al., 2016). Besides, 1D nanostructures with layer-by-layer flakes have been proved favorable to increase the Li^+^ diffusion rate and enlarge the contact areas between electrode and electrolyte (Liang et al., 2013a). layer-by-layer structured K_0.25_V_2_O_5_ electrode exhibits a high initial discharge specific capacity of 256 mA h g^−1^ and superior long-term cycling stability (Fang et al., 2015).

In this work, we reported the synthesis of single crystal fork-like K_2_V_8_O_21_ (KVO) by a facile electrospinning method with a subsequent calcination process. Fork-like K_2_V_8_O_21_ material with layer-by-layer stacking structure has been fabricated and is used as a cathode material in LIBs. The K_2_V_8_O_21_ forks exhibit high specific capacity and superior long-term cycling stability.

## Experimental

### Materials and synthesis

All reagents and solvents were of analytical grade and used as received without further purification. K_2_V_8_O_21_ was synthesized by single-nozzle electrospinning technique with subsequent annealing. Vanadium pentoxide (V_2_O_5_, ≥ 99.0 %), oxalic acid (H_2_C_2_O_4_·2H_2_O, ≥ 98.0 %), potassium nitrate (KNO_3_, ≥ 99.0 %) and polyvinylpyrrolidone (PVP, Mw ≈ 1, 300, 000) were used as starting materials. In a typical synthesis, 0.1 g of V_2_O_5_ and H_2_C_2_O_4_·2H_2_O in a molar ratio of 1:3 were dissolved in 5 mL of de-ionized water (DI) under vigorous stirring at 80°C for about 30 min to form a clear dark blue solution. Then, 0.0278 g of KNO_3_ was added into the above vanadium oxalate solution under magnetic stirring for 10 min before the addition of 5 mL of N,N-Dimethylformamide (DMF) and 1 g of PVP to form a homogeneous viscous dark blue precursor solution. Subsequently, the precursor solution was loaded into a 10 mL plastic syringe with a 21-gauge stainless steel nozzle. The solution was subjected to electrospinning at a DC voltage of 12 kV under a flow rate of 0.04 mm min^−1^. The electrospun nanofibers were collected on aluminum foil with a distance of 15 cm between the nozzle and aluminum collector. Finally, the obtained precursor nanofibers were annealed in a muffle furnace at 500°C in air for 2 h to yield the fork-like K_2_V_8_O_21_. The temperature ramping rate was set of 1°C min^−1^.

### Material characterization

The crystal phase of the as-prepared K_2_V_8_O_21_ were characterized by X-ray diffraction (XRD, Rigaku D/Max 2500) with non-monochromated Cu Kα radiation (λ = 1.54178 Å). The microscopic morphology of the products was investigated by scanning electron microscopy (SEM, FEI Nova Nano SEM 230) and transmission electron microscopy (TEM, JEOL JEM-2100 F). Thermogravimetric analysis (TGA) was carried out on a NETZSCH STA 449C analyzer in air from room temperature to 700°C with a heating rate of 10°C min^−1^.

### Electrochemical measurements

Electrochemical measurement was performed using standard CR2016 type coin cells. The fork-like K_2_V_8_O_21_, acetylene black and polyvinylidene fluoride (PVDF) binder in a weight ratio of 70:20:10 were added into N-methyl-2-pyrrolidone (NMP) solution to make the slurry, which was coated on alumina foil and dried at 100°C overnight under vacuum to obtain the electrodes. The mass loading of the K_2_V_8_O_21_ cathode material for coin cell testing was about 1 mg cm^−2^. All coin cells were assembled in a glove box (Mbraun, Germany) filled with ultra-high pure argon gas. Metallic lithium foils and polypropylene membrane were used as counter electrode and separator, respectively. And 1 M LiPF_6_ solution in ethylene carbonate/dimethyl carbonate (EC/DMC; 1:1, v/v) was used as the electrolyte. The cyclic voltammetry (CV) measurements of Li/K_2_V_8_O_21_ coin cell was conducted using an electrochemical workstation (CHI660C, China) at a scan rate of 0.1 mV s^−1^ in the voltage of 1.5–4 V. The galvanostatic charge/discharge (GCD) performances of the K_2_V_8_O_21_ electrodes were conducted at room temperature on a Land Battery Tester (Land CT2001A, China) in a voltage range of 1.5–4.0 V vs. Li/Li^+^.

## Results and discussion

**Figure 1:**
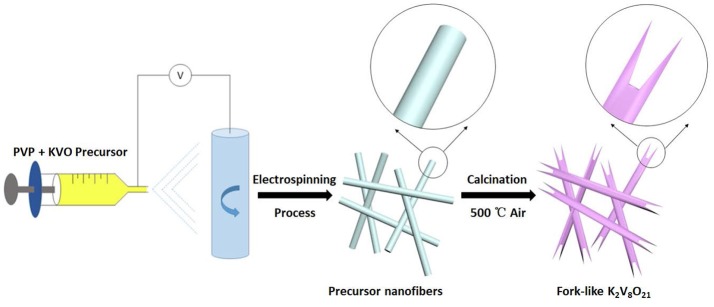
Schematic illustration for the synthesis of fork-like K_2_V_8_O_21_.

Figure 1 shows the synthesis process of fork-like K_2_V_8_O_21_ through the single-nozzle electrospinning technique and a subsequent annealing. Firstly, KNO_3_, V_2_O_5_, H_2_C_2_O_4_·2H_2_O, PVP, DMF and DI were mixed to form a homogeneous viscous precursor, which was electrospun into nanofibers by electrospinning process. The followed annealing treatment can convert the precursor nanofibers to the fork-like crystalline K_2_V_8_O_21_ nanostructures in air at 500°C.

**Figure 2:**
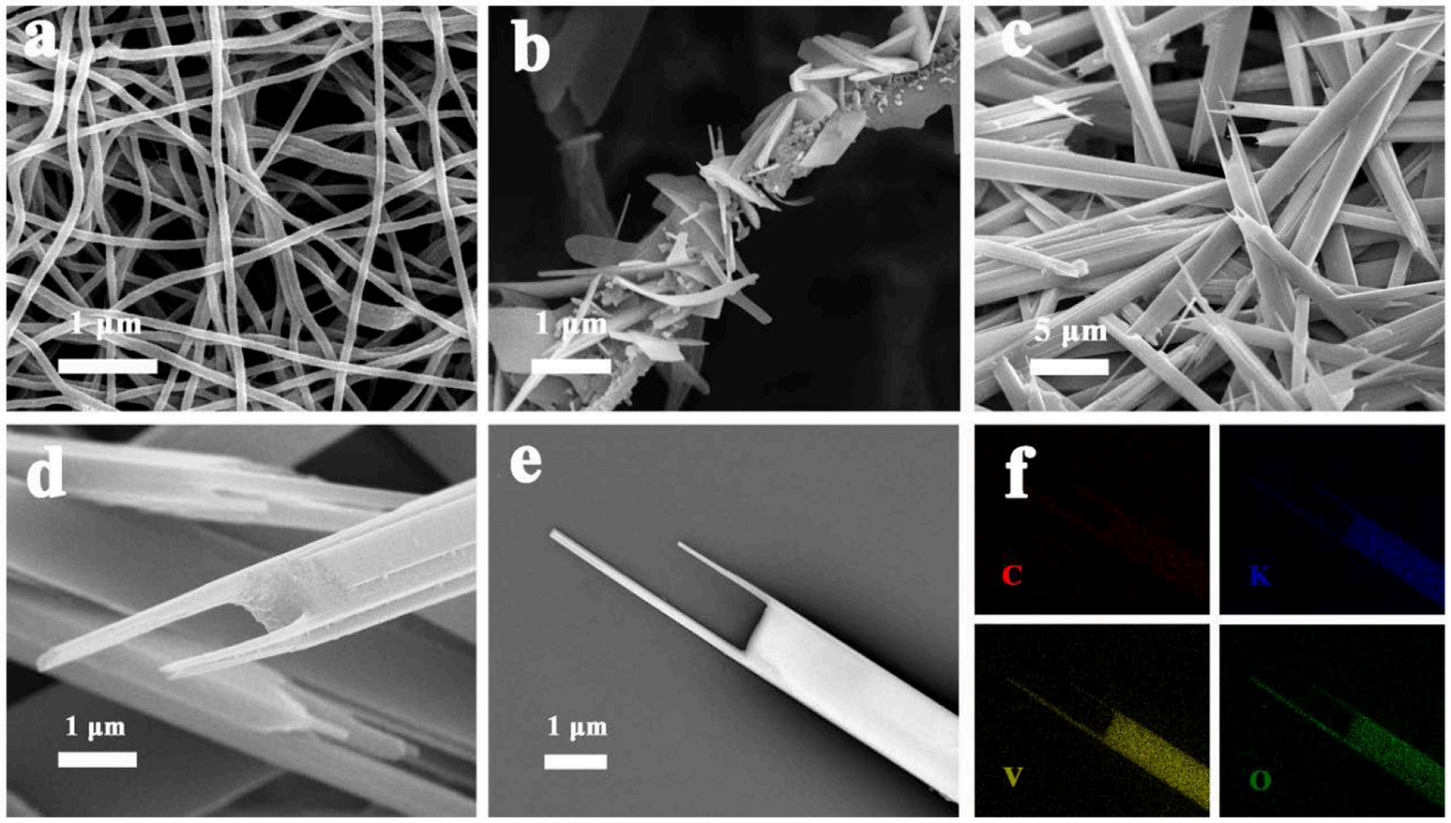
Different magnifications of SEM images of: **(a)** the precursor nanofibers and K_2_V_8_O_21_ nanostructures that were annealed at **(b)** 350°C, **(c–e)** 500°C; **(f)** Elemental mapping images of K_2_V_8_O_21_ compound with the element of C, K, V, O.

Figure 2 shows the structure and morphology of the electrospun precursor and its annealed products of fork-like K_2_V_8_O_21_. Figure 2a shows the SEM image of the precursor nanofibers. The K_2_V_8_O_21_ precursor nanofibers has an average diameter of 100 nm and a smooth and uniform surface. The formation of fiber-like structure can be attributed to the polymers serving as templates during the electrospinning process. Typically, the subsequent annealing could remove the backbones of polymers, and control the morphologies of final products such as nanofibers (Wee et al., 2010), nanowires (Mai et al., 2010b), and nanotubes (Zhao et al., 2015). 350°C was chosen for sintering the precursor nanofibers, and it can be seen from Figure 2b that the fibrous structure was not retained. At this temperature, PVP was decomposed and then converted to carbonaceous species (Liang et al., 2007; Teh et al., 2013). Meanwhile, the KVO nanoparticles that encapsulated in the nanofibers were gradually grown into flat flakes. Because of PVP enclosing the precursor with a fibrous structure and restricting the growth of KVO nanoparticles, KVO small flakes were formed into a hierarchical nanobelt structure. As shown in Figure 2c, the final fork-like K_2_V_8_O_21_ that combined nanorods and nanoflakes formed when the heating temperature was elevated to 500°C. During the annealing process, the transformation of KVO nanoflakes into KVO forks occurred, which was caused by the re-crystallization of K_2_V_8_O_21_. Large space can be clearly seen among the K_2_V_8_O_21_ forks, which may be favorable to the diffusion of lithium ion. Figure 2d shows a single K_2_V_8_O_21_ fork at high magnification, which reveals the layered structures of KVO forks, indicating a feature of layer-by-layer structure. K_2_V_8_O_21_ compound was also prepared by sol-gel route for comparison. The width of sol-gel K_2_V_8_O_21_ compound (SG-KVO) is larger than that of the electrospun fork-like K_2_V_8_O_21_ as shown in Figure S1 (Supplementary Material). Furthermore, the electrospun fork-like K_2_V_8_O_21_ (ES-KVO) show two rod-like tips which are different from the sol-gel K_2_V_8_O_21_, which could be induced by the combustion of PVP and re-crystallization of K_2_V_8_O_21_ clusters. Figure 2f shows the element mapping of fork-like K_2_V_8_O_21_ in Figure 2e, in which the red, blue, yellow and green spot represent the element of carbon, potassium, vanadium and oxygen elements, respectively. It can be seen that these elements are present in the sample and homogeneously dispersed on the fork-like nanostructures, further confirming that C element is well distributed in the fork-like K_2_V_8_O_21_.

**Figure 3:**
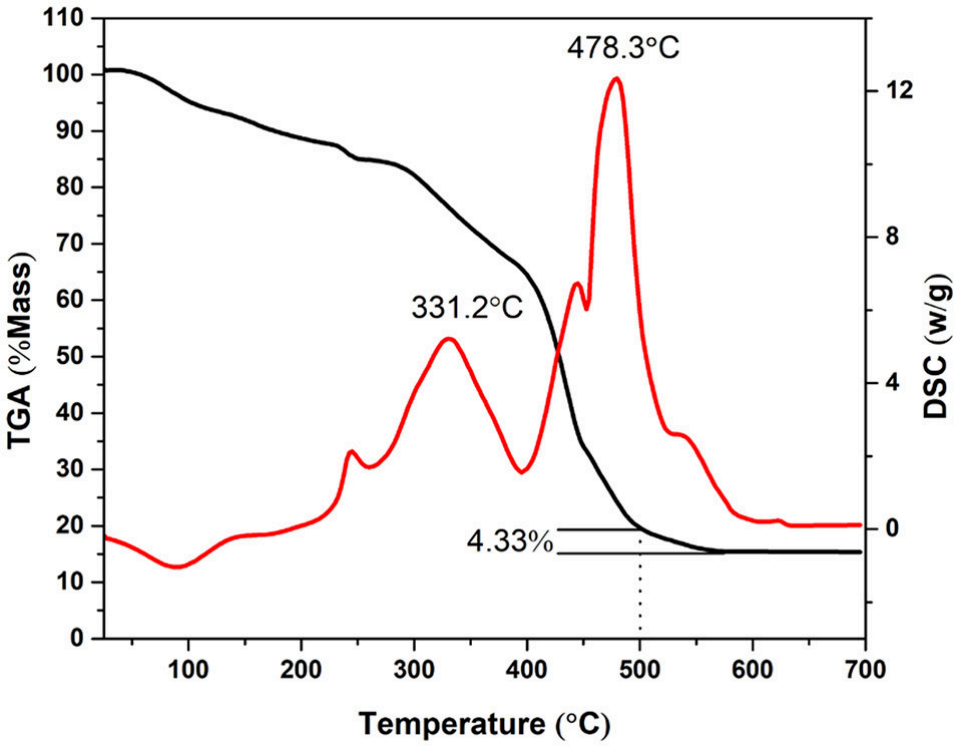
Thermogravimetric analysis results of the KVO precursor nanofibers in air from room temperature to 700°C. The temperature ramping rate was 10°C min^−1^.

Thermogravimetric analysis (TGA) was performed from 25 to 700°C to investigate the thermal decomposition of KVO at a heating rate of 10°C min^−1^ (Figure 3). The as-obtained KVO nanofibers lost mass weight owing to the decomposition of metallic precursor and PVP during the annealing process, leading to the significant surface rupture and the formation of K_2_V_8_O_21_ simultaneously. The mass loss within the range of 25–200°C was attributed to the loss of volatile components, such as residual solvent (DMF, H_2_O) and adsorbed moisture (Ko et al., 2015). At 332.2°C, there was a prominent DSC exothermic peak with a dramatic mass-loss. This exothermic peak may be due to the decomposition of vanadium oxalate, potassium nitrate and the degradation of PVP, which has both intra—and intermolecular transfer reactions according to the degradation mechanisms (Azhari and Dish, 1998). The following exothermic peak, which was accompanied with a significant weight loss at 478.3°C was probably due to the formation of K_2_V_8_O_21_ compound and the oxidation of carbon and carbon monoxide that originated from PVP molecules (Wang et al., 2008). It can be concluded that at 500°C the chemical reaction was completed and the K_2_V_8_O_21_ was obtained. From 500 to 570°C, there was a mass loss about 4.33% and stabilized at about 15% at temperatures above 570°C, which implied that some C elements were remained when the fork-like KVO was obtained.

**Figure 4:**
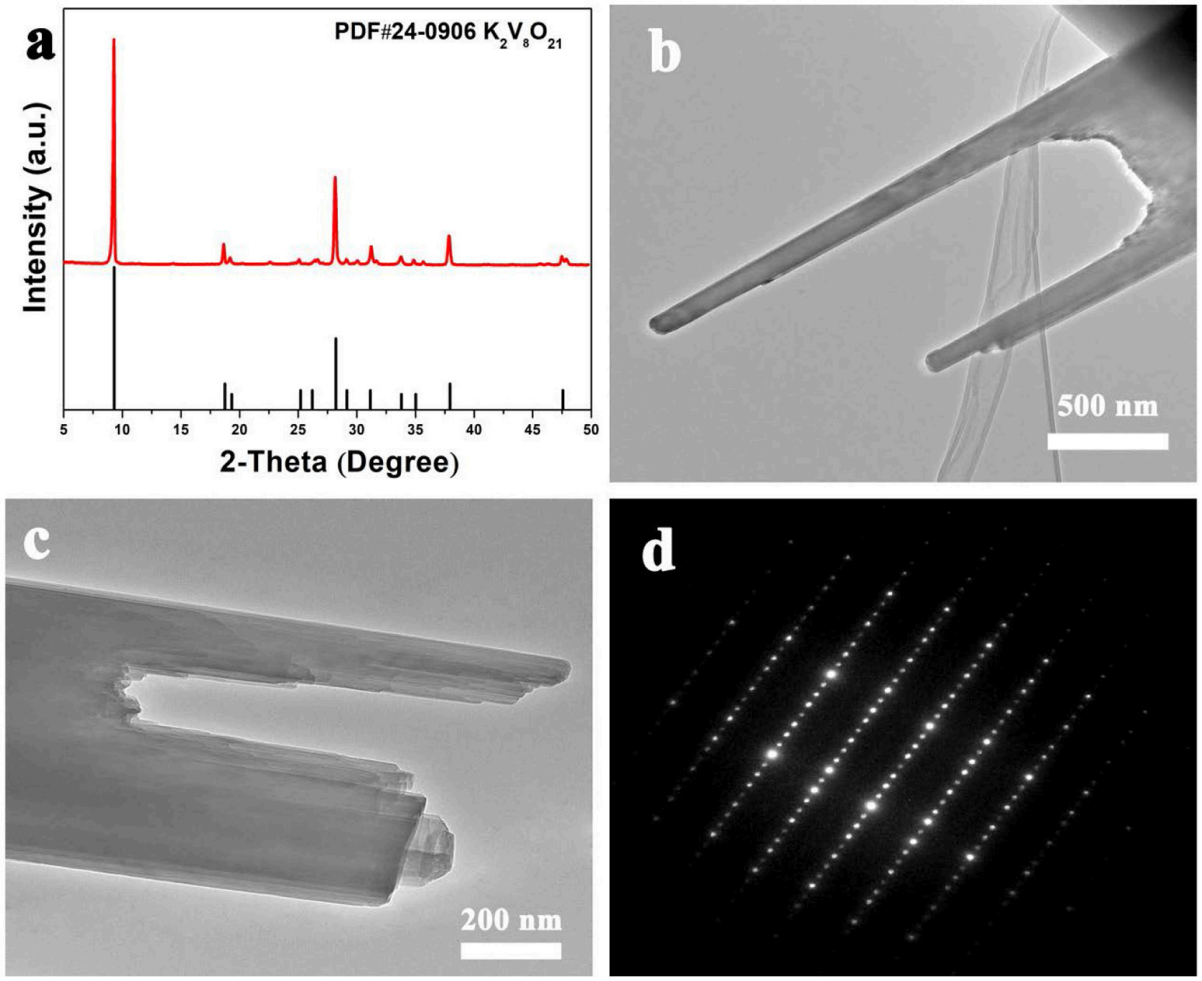
**(a)** XRD patterns, **(b,c)** TEM images and **(d)** SAED patterns of the as-obtained K_2_V_8_O_21_.

The fork-like K_2_V_8_O_21_ prepared *via* electrospinning route was examined by X-ray diffraction and the result is showed in Figure 4a. The as-obtained product shows obvious XRD diffraction peaks, which illustrate the good crystallinity of the product. As for the XRD pattern, all the diffraction peaks show good agreement with K_2_V_8_O_21_ phase (JCPDS Card No. 24-0906). No obvious peaks from other phases can be found, demonstrating that the as-prepared K_2_V_8_O_21_ is of high purity. It is worth noting that the diffraction pattern of K_2_V_8_O_21_ has not been fully studied so far since the structure of this material was found.

The structure of the as-prepared fork-like K_2_V_8_O_21_ was further examined by transmission electron microscopy (TEM). The TEM image in Figure 4b shows the morphology of the endpoint of the obtained K_2_V_8_O_21_. It can be confirmed that the fork-like nanostructure is consistent with the SEM observation (Figure 2c). As is shown in Figure 4c, a detailed examination of a KVO folk reveals the broken tips of the K_2_V_8_O_21_ folk and the endpoint of K_2_V_8_O_21_ are transparent under the electron beam, implying an ultrathin property of the nanorods. Moreover, it is distinct that the nanorods exhibit layer-by-layer stacked structures, which is beneficial to the lithium intercalation and de-intercalation during the charge-discharge process (Pan et al., 2011b). The selected area electron diffraction (SAED) pattern is displayed in Figure 4d, which indicates that the as-prepared K_2_V_8_O_21_ nanorods are single-crystalline which is consistent with a previous report (An et al., 2010). It is generally accepted that single-crystalline nanostructured materials hold no significant grain boundaries with few defects, which facilitates the Li-ion diffusion during electrochemical reactions because Li ions need not to go across grain boundary and defects (Liang et al., 2007; Chen, 2013).

**Figure 5:**
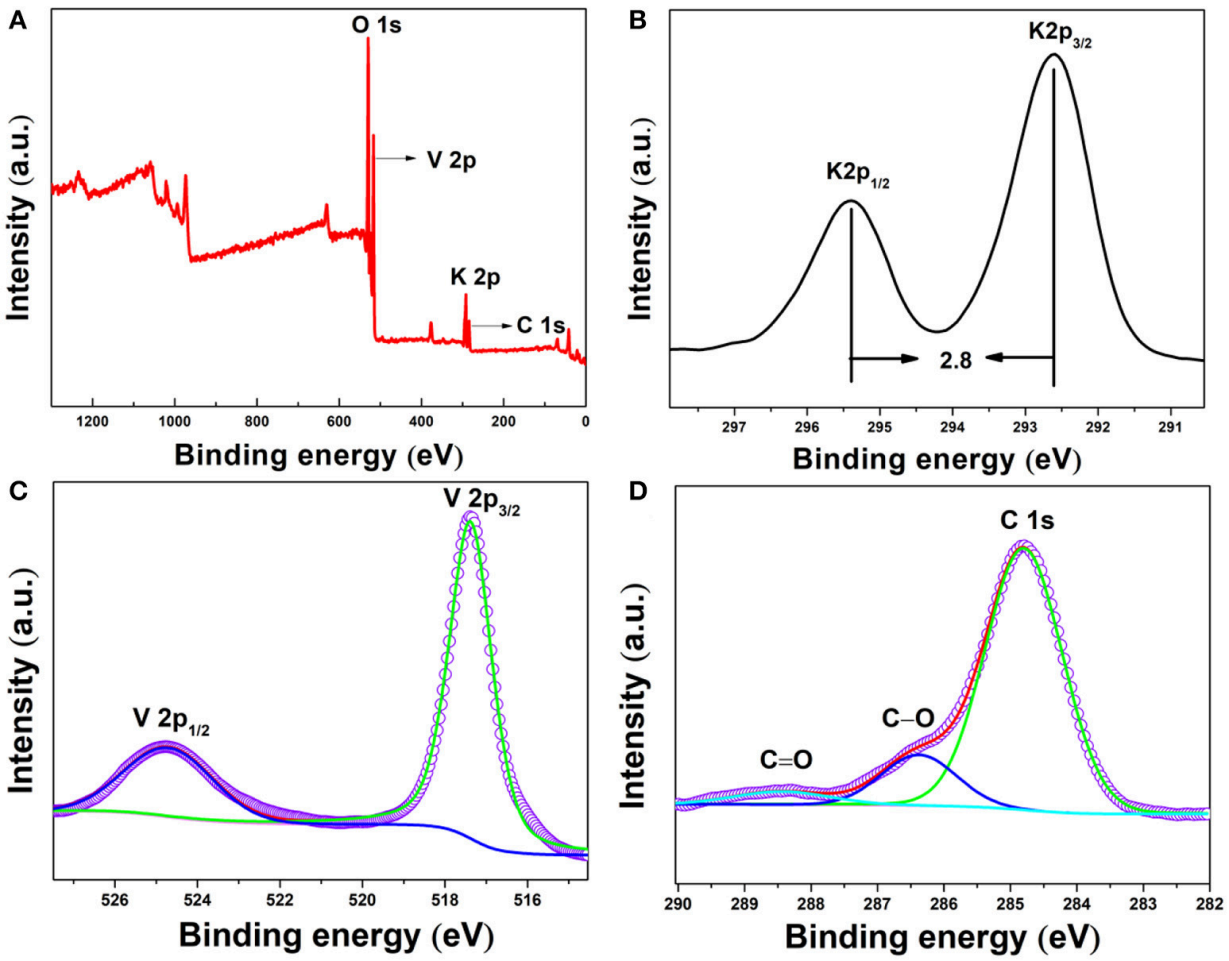
XPS spectra for K_2_V_8_O_21_ compound: **(A)** survey scan spectrum and high-resolution spectra of **(B)** K 2p, **(C)** V 2p and **(D)** C 1s.

X-ray photoelectron spectroscopy (XPS) was further conducted to prove the chemical components and element valences of fork-like K_2_V_8_O_21_. As shown in Figure 5A, full survey spectrum reveals the existence of potassium, vanadium, oxygen and carbon elements in the as-prepared product. The peaks at 530.2, 517.2, 292.6, and 284.8 eV can be ascribed to O 1s, V 2p_3/2_, K 2p_3/2_, and C 1s, respectively. Correspondingly, the peaks at 292.6 and 295.4 eV which are 2.8 eV apart in the high-resolution spectrum of K 2p (Figure 5B) could be ascribed to K 2p_3/2_ and K 2p_1/2_ of K^+^ (Li et al., 2001). As shown in Figure 5C, the characteristic doublet of potassium vanadate V 2p is found at 517.6 and 524.7 eV, revealing that the fork-like K_2_V_8_O_21_ only contains V^5+^ valence state and no V^4+^ can be detected (Silversmit et al., 2004). In the broad spectrum (Figure 5A), some C element was observed and in the high-resolution spectrum of C 1s (Figure 5D), the peaks centered at 284.8, 286.4 and 288.5 eV were due to the C-C, C-O and C = O bonds, respectively. The existence of C element derived from PVP was partially retained after annealing.

**Figure 6:**
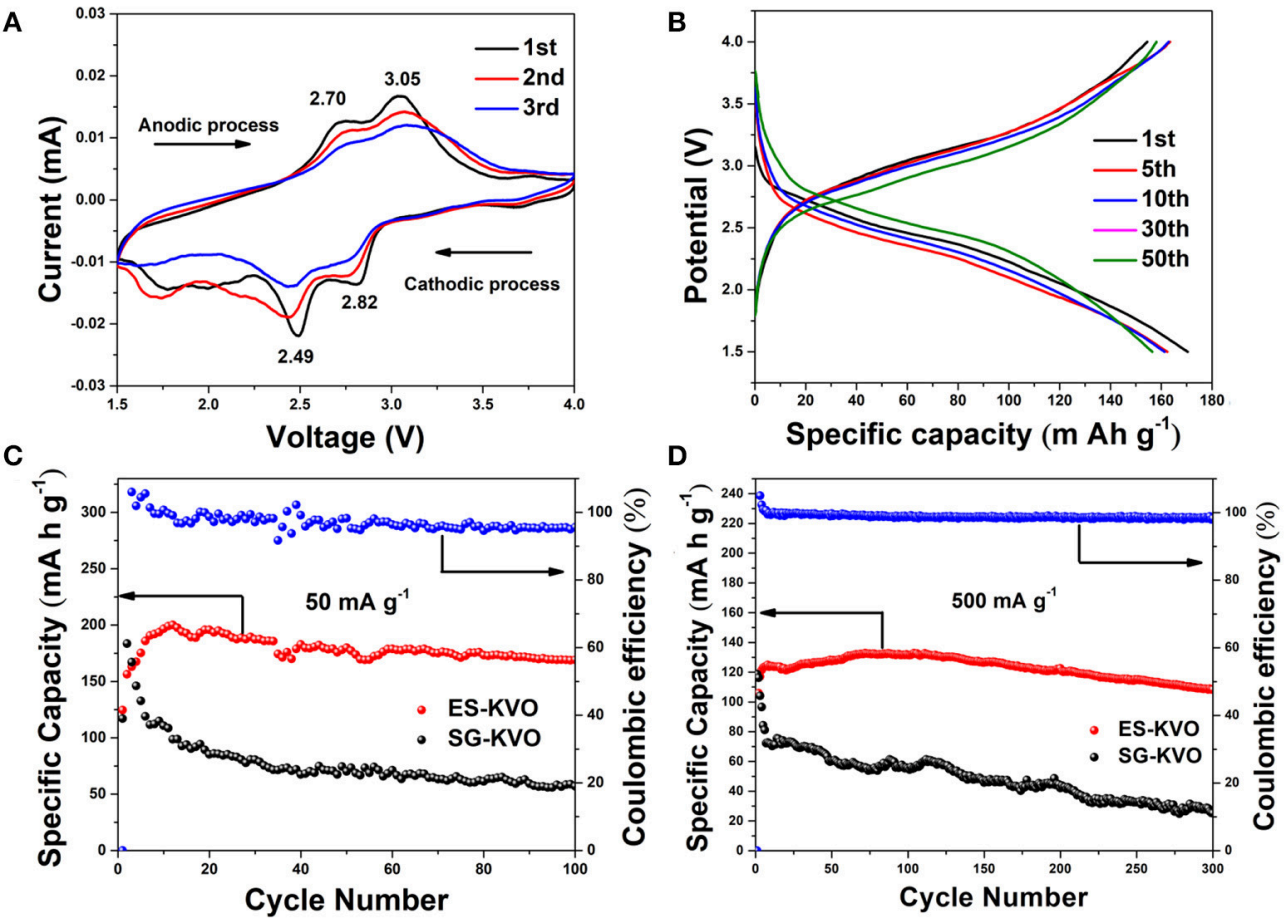
**(A)** The first three CV curves at a scan rate of 0.1 mV s^−1^ within a voltage range of 1.5–4.0 V and **(B)** GCD profiles of 1st, 5th, 10th, 30^th^, and 50th cycle at 100 mA g^−1^ of the K_2_V_8_O_21_ electrode; the cycling performances of ES-KVO and SG-KVO electrodes at the current density of **(C)** 50 mA g^−1^ and **(D)** 500 mA g^−1^.

The electrochemical properties of the electrospun fork-like K_2_V_8_O_21_ as cathode are evaluated by assembling CR2016 type coin half lithium-ion batteries. The cyclic voltammetry (CV) curves of K_2_V_8_O_21_ electrode is recorded in a voltage window of 1.5–4.0 V at a scan rate of 0.1 mV s^−1^. As shown in Figure 6A, two main reduction peaks located at 2.49 V and 2.82 V are ascribed to the intercalation of the lithium ions into K_2_V_8_O_21_ electrode in several steps. Moreover, a broad peak at about 1.75 V is also detected, which suggest further intercalation of lithium ions into the electrode material. During the anodic scan, two main oxidation peaks near 2.70 V and 3.05 V are well detected, which can be attributed to the de-intercalation of the lithium ions (Manev et al., 1993). According to the three consecutive cycles, the electrode materials has the structural reversibility due to the redox peak positions do not change much, although the peak current intensity decreases gradually. The intercalation/de-intercalation behavior of lithium in the cycling process of K_2_V_8_O_21_ electrode can be expressed as equation (1):

(1) $$\begin{array}{r} {\text{K}}_{2}{\text{V}}_{8}{\text{O}}_{21}+\text{x}\text{L}{\text{i}}^{+}+\text{x}{\text{e}}^{-}⇌\text{L}{\text{i}}_{\text{x}}{\text{K}}_{2}{\text{V}}_{8}{\text{O}}_{21} \end{array}$$

Based on the previous reports on the voltage window and CV curves (Figure 6A), 1.5–4 V was chose as voltage window (Ni et al., 2015). Figure 6B shows the typical charge-discharge voltage profiles of K_2_V_8_O_21_ electrode and enumerates the 1st, 5th, 10th, 30th, and 50th cycles at a constant current density of 100 mA g^−1^. There are two voltage plateaus in both charge and discharge curves as shown in Figure 6B. In discharge process, there are two discharge plateaus at around 2.5 and 2.8 V, respectively, which are consistent with the previous CV curves (Figure 6A) that is due to the lithium-ion intercalation process. These charge/discharge profiles in different cycles have shown similar shapes, which demonstrate the good structural stability of this material. It is worth noting that, although the capacity fades upon cycling, the overpotentials on both charging and discharging are reduced, this can compensate the loss in the energy density upon cycling. So the energy efficiency of the 5th, 10th, 15th, 30th, and 50th cycles at a constant current density of 100 mA g^−1^ was calculated according to the following equation (2) (Eftekhari, 2017):

(2) $$\begin{array}{r} \text{Energy efficiency}=\frac{\text{energy density }\left(\text{discharge}\right)}{\text{energy density }\left(\text{charge}\right)}\times 100\% \end{array}$$

At various cycles of 5th, 10th, 15th, 30th, and 50th, the electrode exhibits energy efficiencies of 69.19, 71.15, 74.52, 77.55, and 78.02%, respectively. The improvement of Li^+^ diffusion may cause the energy efficiency increase during the cycling process, which is beneficial to use K_2_V_8_O_21_ as a cathode material. Figure 6C displays the cycling performance and coulombic efficiency of the electrospun K_2_V_8_O_21_ and sol-gel K_2_V_8_O_21_ at 50 mA g^−1^, respectively. An initial specific discharge capacity of 124.6 mA h g^−1^ is obtained from ES-KVO electrode and then the capacity increased gradually to reach a maximum value of 200.2 mA h g^−1^ at the 12th cycle. The activation process and the wetting of active electrode at the beginning may give rise to a capacity increase. After 100 cycles, the ES-KVO cathode still retains a discharge capacity of 169.2 mA h g^−1^ with an average capacity fading rate of 0.037% per cycle based on the discharge capacity of 5th cycle. Furthermore, the coulombic efficiency can be maintained above 95%. For comparison, the SG-KVO electrode can only deliver a maximum specific discharge capacity of 183.6 mA h g^−1^ and fade rapidly to <100 mA h g^−1^. It can be demonstrated from the above results that the as-obtained electrospun K_2_V_8_O_21_ electrode has shown a better cycling stability.

The long-term cycling performance of K_2_V_8_O_21_ electrode is also examined. The long-term cycling performance and coulombic efficiency of electrospun K_2_V_8_O_21_ and sol-gel K_2_V_8_O_21_ electrode at a same current density of 500 mA g^−1^ are shown in Figure 6D. Similar to the cycling performances performed at 50 mA g^−1^, the capacity of ES-KVO electrode increased in the initial several cycles at 500 mA g^−1^. This may be caused by the activation at the beginning and the improvement of lithium ion accessibility in the electrode materials during the cycling process. Although the initial discharge capacity of the cell is 120.8 mA h g^−1^, then the specific capacity increases slowly to reach a maximum value of 131.5 mA h g^−1^ at 72th cycle. After 300 cycles, a discharge capacity of 108.3 mA h g^−1^ still can be retained with a fading rate of only 0.043 % per cycle based on the capacity of 4th cycle. Besides, a high coulombic efficiency ~98% can be reached throughout the cycling, which shows a good reversibility of this K_2_V_8_O_21_ electrode at a high current rate. The initial coulombic efficiency of the K_2_V_8_O_21_ cathode was 114.07 %, which may be caused by the partial removal of K^+^ ions from the electrode materials during the charge process at initial cycle. An initial discharge capacity of SG-KVO electrode about 118.7 mA h g^−1^ is obtained but then dropping rapidly for subsequent cycles. After 300 cycles, the sol-gel electrode can only deliver a low discharge capacity of 25.2 mA h g^−1^, showing a poor long-term cycling stability. The fork-like K_2_V_8_O_21_ prepared by electrospinning has displayed superior specific capacity and long-term cycling stability compared to the SG-KVO, implying that it is a promising candidate for lithium-ion batteries as a cathode material. Moreover, Table S1 (Supplementary Material) summaries many reported electrochemical performance of metal vanadium oxides. Based on the comparison result, the reported fork-like K_2_V_8_O_21_ in this work shows excellent cycle performance when it was used as a cathode material for LIBs.

As discussed above, the electrospun fork-like K_2_V_8_O_21_ has demonstrated superior electrochemical performance, which may be attributed to the following reasons: (1) the morphology of layer-by-layer stacked fork-like nanostructure is propitious to lithium-ion diffusion and has enlarged the contact area between electrode and electrolyte; (2) the large space between the fork-like K_2_V_8_O_21_ could promote the diffusion of lithium-ion and buffer the volume change during the electrochemical reactions; (3) the single crystalline property of K_2_V_8_O_21_ can offer convenient pathways for lithium ion diffusion to increase the efficiency of lithiation and delithiation.

## Conclusions

In summary, we have successfully prepared a single crystalline fork-like K_2_V_8_O_21_ *via* facile electrospinning method followed by an annealing process in air at 500°C for 2 h. The single crystalline fork-like K_2_V_8_O_21_ has shown layer-by-layer stacked nanostructure with large space and conductive carbon through heat treatment of KVO precursor. Due to this advantageous feature, the fork-like K_2_V_8_O_21_ demonstrates excellent electrochemical performances including high specific discharge capacity of 200.2 mA h g^−1^ at a current density of 50 mA g^−1^ with a good capacity retention (96.52%) after 100 cycles. Moreover, the electrodes demonstrate superior long-term cycling stability up to 300 cycles at a current density of 500 mA g^−1^. The results from our work have demonstrated that the as-electrospun K_2_V_8_O_21_ is a promising cathode candidate for next-generation high-performance LIBs.

## Author contributions

PH did the main experiment and write the manuscript. TZ involved in the discussion of the experiment and revised the manuscript. QS and JL did the SEM experiment. RC assisted the material synthesis. XC, YW, and AP made the research plan. AP also provided the financial support.

### Conflict of interest statement

The authors declare that the research was conducted in the absence of any commercial or financial relationships that could be construed as a potential conflict of interest.
