# Supplementary material for: Electrospun Single Crystalline Fork-Like K2V8O21 as High-Performance Cathode Materials for Lithium-Ion Batteries
Source: Front Chem. 2018 Jun 1;6:195. doi: 10.3389/fchem.2018.00195 (PMC5992297; doi:10.3389/fchem.2018.00195)
Supplement: Supplementary file 1 [file Presentation_1.pdf]

Supplementary Material

Electrospun single crystalline fork-like $K_2V_8O_{21}$ as high-performance cathode materials for lithium-ion batteries

Pengfei Hao, Ting Zhu, Qiong Su, Jiande Lin, Rong Cui, Xinxin Cao, Yaping Wang, Anqiang Pan*

*** Correspondence:** Anqiang Pan：pananqiang@csu.edu.cn

1 Supplementary Figures and Tables

1.1 Supplementary Figures

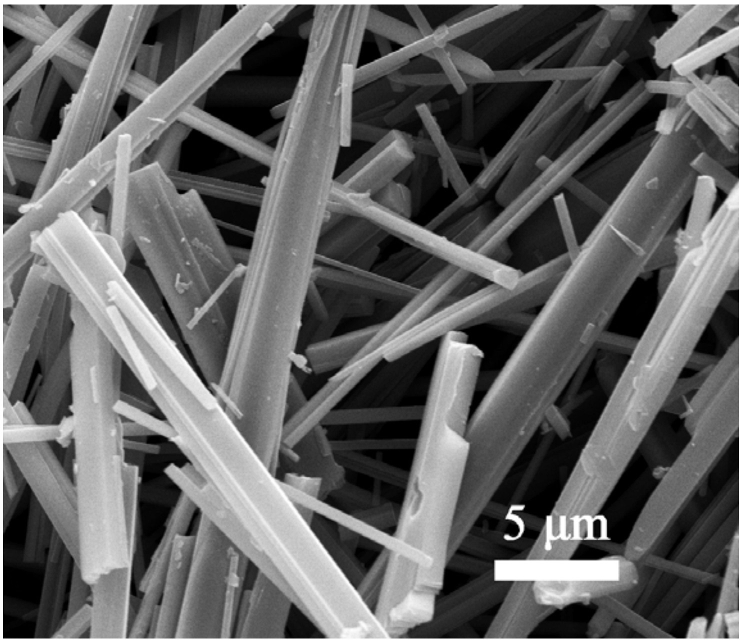

Fig. S1. SEM image of sol-gel $K_2V_8O_{21}$ that were annealed at 500 ˚C

1.2 Supplementary Tables

Table S1. Comparison on electrochemical performance of fork-like $K_2V_8O_{21}$ and other metal vanadium oxides

Synthesis method (ref)	Sample	Current density (mA g^{-1})	Capacity (mA h g^{-1}) (cycle number)	Fading rate (%, per cycle)
Spray-drying technique(Tran et al., 2008)	$Li_{1.1}V_3O_8$	C/3	260 (2) - 220 (60)	0.26
Solid-state reaction(Wang et al., 2012)	LiV_3O_8	100	282 (1) - 228 (20)	0.95
Solvothermal(Tan et al., 2015)	β-$Na_{0.33}V_2O_5$	1000	157 (1) – 111 (35)	0.84
Hydrothermal(Yang et al., 2016)	$Na_{0.76}V_6O_{15}$	300	247.5 (2) - 222.1 (50)	0.21
Facile synthesis route(Zhou et al., 2014)	Ag/$Ag_{1.2}V_3O_8$	100	190 (2) – 164 (50)	0.28
Solid-state reaction(Liang et al., 2013)	Ag/$AgVO_3$	50	243 (1) – 111 (30)	1.81
Hydrothermal(Xu et al., 2015)	$K_{0.23}V_2O_5$	50	243 (1) - 185 (100)	0.24
Solution route(Baddour-Hadjean et al., 2014)	$K_{0.5}V_2O_5$	C/10	220 (10) – 200 (70)	0.15
	$K_{0.25}V_2O_5$	C/10	230 (1) – 153 (70)	0.48
Sol-gel route(Fang et al., 2015)	$K_{0.25}V_2O_5$	100	211 (1) – 189 (50)	0.21
		500	131 (3) – 116 (500)	0.023
Hydrothermal(Huang et al., 2014)	$K_{0.66}V_3O_8$	75	197.5 (1)-139.8 (25)	1.17
Solid-state reaction(Aleksandrova et al., 2009)	$K_2V_8O_{21}$	C/3	197 (1) – 170 (50)	0.28
Solid-state reaction(Ni et al., 2015)	$K_2V_8O_{21}$	100	213 (1) -160 (20)	1.25
Electrospinning method (this work)	$K_2V_8O_{21}$	50	200.2 (12) – 169.2 (100)	0.18
		500	124.2 (4) – 108.3 (300)	0.043

References

Aleksandrova, A., Uzunov, I., Banov, B., and Momchilov, A. (2009). Potassium bronzes as active material for Li-ion batteries. Compt. rend. Acad. bulg. 62, 453-460.

Baddour-Hadjean, R., Boudaoud, A., Bach, S., Emery, N., and Pereira-Ramos, J.P. (2014). A comparative insight of potassium vanadates as positive electrode materials for Li batteries: influence of the long-range and local structure. *Inorg. Chem.* 53**,** 1764-1772. doi: 10.1021/ic402897d

Fang, G., Zhou, J., Hu, Y., Cao, X., Tang, Y., and Liang, S. (2015). Facile synthesis of potassium vanadate cathode material with superior cycling stability for lithium ion batteries. J. Power Sources 275, 694-701. doi: 10.1016/j.jpowsour.2014.11.052

Huang, X., Dai, B., Xu, G., He, X., Zhou, S., Chen, Y., and Liu, B. (2014). Potassium vanadate nanobelt as a high-capacity cathode material for li-ion battery. *Int. J. Electrochem. Sci.* 9**,** 6640-6647.

Liang, S., Zhou, J., Pan, A., Zhang, X., Tang, Y., Tan, X., Chen, T., and Wu, R. (2013b). Facile synthesis of Ag/$AgVO_3$ hybrid nanorods with enhanced electrochemical performance as cathode material for lithium batteries. *J. Power Sources* 228**,** 178-184. doi: 10.1016/j.jpowsour.2012.11.104

Ni, E.F., Goto, S., Quan, Z., and Sonoyama, N. (2015). Electrochemical Property for the Metal-doped Vanadium Bronze $K_2V_8O_{21}$ as a Cathode Material of Lithium Battery. *Electrochemistry* 83**,** 902-908. doi: 10.5796/electrochemistry.83.902

Tan, Q., Zhu, Q., Pan, A., Wang, Y., Tang, Y., Tan, X., Liang, S., and Cao, G. (2015). Template-free synthesis of β-$Na_{0.33}V_2O_5$ microspheres as cathode materials for lithium-ion batteries. *CrystEngComm* 17**,** 4774-4780. doi: 10.1039/c5ce00635j

Tran, N., Bramnik, K.G., Hibst, H., PröLß, J., Mronga, N., Holzapfel, M., Scheifele, W., and NováK, P. (2008). Spray-Drying Synthesis and Electrochemical Performance of Lithium Vanadates as Positive Electrode Materials for Lithium Batteries. *J. Electrochem. Soc.* 155**,** A384. doi: 10.1149/1.2884859

Wang, D., Cao, L., Huang, J., and Wu, J. (2012). Synthesis and electrochemical properties of submicron sized sheet-like LiV_3O_8 crystallites for lithium secondary batteries. *Mater. Lett.* 71**,** 48-50. doi: 10.1016/j.matlet.2011.12.019

Xu, M., Han, J., Li, G., Niu, Y., Liu, S., Hou, J., Wang, M., Song, J., and Li, C.M. (2015). Synthesis of novel book-like $K_{0.23}V_2O_5$ crystals and their electrochemical behavior in lithium batteries. *Chem. Commun.* 51**,** 15290-15293. doi: 10.1039/c5cc05425g

Yang, K., Fang, G., Zhou, J., Qin, M., Tang, Y., Pan, A., and Liang, S. (2016). Hydrothermal synthesis of sodium vanadate nanobelts as high-performance cathode materials for lithium batteries. *J. Power Sources* 325**,** 383-390. doi: 10.1016/j.jpowsour.2016.06.023

Zhou, J., Liang, Q., Pan, A., Zhang, X., Zhu, Q., Liang, S., and Cao, G. (2014). The general synthesis of Ag nanoparticles anchored on silver vanadium oxides: towards high performance cathodes for lithium-ion batteries. *J. Mater. Chem. A* 2**,** 11029-11034. doi: 10.1039/c4ta00437j
